# A service evaluation of FODMAP restriction, FODMAP reintroduction and long‐term follow‐up in the dietary management of irritable bowel syndrome

**DOI:** 10.1111/jhn.13393

**Published:** 2024-11-05

**Authors:** Rosie Foulkes, Paru Shah, Alice Twomey, Lara Dami, Danielle Jones, Miranda C. E. Lomer

**Affiliations:** ^1^ Department of Nutritional Sciences King's College London London UK; ^2^ Department of Nutrition and Dietetics Guy's and St Thomas' NHS Foundation Trust London UK

**Keywords:** diet, irritable bowel syndrome, low FODMAP

## Abstract

**Background:**

The dietary restriction of fermentable oligosaccharides, disaccharides, monosaccharides and polyols (FODMAPs), called the low‐FODMAP diet (LFD), is frequently used to manage irritable bowel syndrome (IBS). This service evaluation aimed to assess the long‐term effectiveness of the LFD in managing IBS symptoms and whether symptom response and dietary adherence to the LFD were associated.

**Methods:**

This observational service evaluation collected data via questionnaires during clinical dietetic appointments for IBS management. Symptom severity was reported at baseline, short term (following FODMAP restriction) and long term (following FODMAP reintroduction). Additional data that captured experiences following the LFD were collected at long‐term follow‐up.

**Results:**

Of 184 patients, 14% reported satisfactory relief from global symptoms at baseline, which increased to 69% at short‐term follow‐up and 57% at long‐term follow‐up (*p* < 0.001). The most notable improvements in individual symptoms between baseline and long‐term follow‐up were abdominal bloating (72% baseline, 48% long term, *p* < 0.001), abdominal pain (61% baseline, 30% long term, *p* < 0.001) and flatulence (71% baseline, 40% long term, *p* < 0.001). High adherence with the LFD at short‐term follow‐up was not associated with long‐term symptom improvement, but there was an association between long‐term adherence and global symptom severity (*p* = 0.032). Completion of FODMAP reintroduction as per protocol was associated with long‐term symptom improvement (*p* = 0.049).

**Conclusions:**

The LFD is an effective treatment for managing IBS symptoms in the long term, particularly, when the diet is adhered to and reintroduction is completed as per dietetic education. Further randomised‐controlled trials are required to explore the cause‐and‐effect relationship between LFD and IBS symptom management.

## Key points

- The low‐fermentable oligosaccharide, disaccharide, monosaccharide and polyol (FODMAP) diet is an effective treatment strategy for managing symptoms of irritable bowel syndrome in the long term.
- The low‐FODMAP diet is successful when it is adhered to and FODMAP reintroduction is completed according to dietetic education.
- Fructan‐containing foods (onion, garlic, wheat bread) are the most common food challenges during FODMAP reintroduction and are well tolerated in over half of patients who challenge with these foods.

## INTRODUCTION

Irritable bowel syndrome (IBS) is a debilitating disorder of gut–brain interaction (DGBI) characterised by recurrent abdominal pain associated with defecation or altered bowel habits.^1^ Affecting approximately 5% of the population,^2^, ^3^ IBS places an economic burden on society through increased healthcare utilisation and work absenteeism^4^ and negatively impacts quality of life.^5^ The pathophysiology of IBS is complex and involves the dysregulation of several physiological pathways.^6^ Over 80% of patients report symptoms being triggered by dietary factors; therefore, dietary management is an important clinical strategy in managing IBS.^7^

In recent years, the dietary restriction of fermentable oligosaccharides, disaccharides, monosaccharides and polyols (FODMAPs), called the low‐FODMAP diet (LFD), has been studied for its effect on reducing IBS symptoms.^8^ Evidence suggests that FODMAP restriction improves symptoms in 50%–80% of patients.^9^ However, there are concerns regarding the nutritional impact of this restriction in the long term, such as an increased risk of calcium deficiency related to the restriction of dairy products^10^ or reductions in the concentration of beneficial colonic bifidobacteria due to restricting fermentable short‐chain carbohydrates.^11^ Therefore, reintroducing FODMAPs to tolerance is advised, allowing patients to identify their personal threshold for individual FODMAPs and develop a modified LFD that minimises symptoms while maximising dietary diversity.^12^

The LFD is included in guidelines as a second‐line intervention.^13^ Delivery is dietitian‐led either individually or to groups. A first appointment outlines the restriction phase, where high‐FODMAP foods are reduced in the diet for 4–8 weeks, and a second appointment outlines the reintroduction phase, where FODMAPs are systematically reintroduced into the diet to tolerance. A recent systematic review and meta‐analysis comparing the LFD against alternative dietary advice for IBS found that the LFD was more effective at reducing global IBS symptoms compared to all five other interventions studied.^14^ However, few studies examined the long‐term effects of FODMAP reintroduction or personalisation.

One study collected data from primary and secondary care via postal questionnaires 6–18 months after short‐term follow‐up^12^ and found that 57% of patients who received LFD education reported long‐term satisfactory symptom relief, with 82% following a modified LFD at long‐term follow‐up. Furthermore, the modified LFD was found to be nutritionally adequate, acceptable to follow and did not negatively impact food‐related quality of life. However, only 27% (*n* = 103) of the original sample completed the questionnaire; therefore, the results may be subject to nonresponse bias. Another questionnaire study from secondary care found that 60% of patients reported adequate long‐term symptom relief, with 76% following a modified LFD at long‐term follow‐up.^15^ However, this study also had a low response rate of 31% (*n* = 205) and baseline symptom data were not available for all patients, meaning that the results may not be representative of the entire cohort. A recent observational service evaluation recorded IBS symptoms in a dietetic‐led gastroenterology service in primary care specialising in IBS patient appointments before and immediately after FODMAP intervention and collected long‐term data at least 11 months later via a postal questionnaire. Of the patients who responded, 10% reported satisfactory relief from symptoms at baseline, which increased significantly to 55% by long‐term follow‐up.^16^

Some studies measured adherence with the LFD using Likert scales and found that higher adherence was associated with reduced symptom severity.^12^, ^15^ An Irish retrospective cohort study investigating the effects of the LFD over 12 months measured adherence at each appointment using comprehensive food and symptom diaries and dietitian‐led dietary recall. Seventy‐six percent (*n* = 97) of patients fully complied with the LFD at the 3‐month follow‐up, and these patients showed better symptom improvement than those with partial adherence.^17^

Patients who achieve symptom improvement following FODMAP restriction may be hesitant to complete FODMAP reintroduction for fear of symptom recurrence^17^; however, lengthy restrictive diets raise nutritional concerns.^11^, ^18^ Indeed, in one study, only 11% of participants (*n* = 14) agreed to reintroduce high‐FODMAP foods, and yet, those who did all maintained satisfactory symptomatic relief in the long term.^17^ However, in another study, 76% of participants followed a modified LFD at long‐term follow‐up.^15^

The aim of this service evaluation was to evaluate the long‐term efficacy of the LFD in managing IBS symptoms and whether symptom response and dietary adherence to the LFD were associated.

## METHODS

### Study design

The study was a prospective service evaluation of long‐term follow‐up after LFD education in patients with IBS referred from primary and secondary care to a specialist NHS gastroenterology dietetic service between July 2017 and July 2020. Referrers were not aware of the service evaluation. Patients received three clinical appointments with symptom questionnaires collected at each appointment as part of usual clinical care and described elsewhere.^8^ The initial appointment provided dietary counselling and resources for FODMAP restriction (baseline); the second appointment was four to eight weeks later and educated patients on FODMAP reintroduction (short‐term follow‐up); and the third appointment was 6–18 months later (long‐term follow‐up). At long‐term follow‐up, additional questions regarding individual experiences following the LFD were collected. Baseline and short‐term follow‐up appointments were delivered as one‐to‐one or group education in person (baseline and short‐term follow‐up) or by telephone (short‐term follow‐up or long‐term follow‐up).

### Eligibility, ethics and consent

Patients with IBS according to standard clinical criteria who had received education on the LFD were eligible to participate. Patients were excluded from analysis if they did not attend an appointment or their questionnaire was incomplete for symptom data for at least one of the appointments.

Ethical approval was not required and the study was approved as a service evaluation (reference 11657). Clinical consent was obtained as part of routine clinical care.

### Data collection and outcomes

Anonymised demographic, clinical, symptom and dietary data were extracted into a purpose‐built database directly from the patients' electronic clinical record by several researchers.

#### Gastrointestinal symptoms and stool output

The global symptom question (GSS) “Do you currently have satisfactory relief of your gut symptoms?” and validated Gastrointestinal Symptom Rating Scale^19^ were used to rate the severity of individual IBS symptoms over the previous week via a four‐point Likert scale (none, mild, moderate, severe). Where more than one option was ticked, the more severe option was recorded. Data for stool frequency and consistency (Bristol stool form scale) were collected as the best description of bowel activity over the previous week, with patients allowed to select multiple options if necessary.

#### Adherence

At short‐term follow‐up, a five‐point Likert scale was used to rate the percentage of time spent following the diet (0%, 25%, 50%, 75%, 100%), indicating how strictly patients adhered to the LFD during FODMAP restriction. Adherence was where patients reported following the LFD 75%–100% of the time and nonadherence was where patients reported following the LFD less than 75% of the time.

At long‐term follow‐up, patients recorded the most appropriate phase of the LFD diet that they were currently following (‘continues on strict LFD’, ‘FODMAP reintroduction’, ‘modified LFD’, i.e., patients had reintroduced FODMAPs in accordance with the protocol, ‘returned to habitual diet’). Adherence was defined as patients who reported a modified LFD at long‐term follow‐up and non‐adherence was defined as patients who reported any of the following: continues on strict LFD; FODMAP reintroduction; and returned to habitual diet.

#### FODMAP reintroduction and long‐term data

At long‐term follow‐up, patients were asked to record which high‐FODMAP foods they had challenged during FODMAP reintroduction and whether they had or had not tolerated the food. Each food was grouped into FODMAP categories: fructans, galacto‐oligosaccharides, lactose, fructose, sorbitol, mannitol and more than one FODMAP.

Patients were asked if they had completed FODMAP reintroduction (yes, no, partial), if they had identified any nondietary factors contributing to symptoms (yes, no), if they were using nondietary interventions to manage symptoms (yes, no), whether the diet helped, hindered or had no effect on social situations, how they found the reintroduction instructions (difficult, okay, easy), if they had identified any difficulties carrying out the challenges (yes, no), with comments on the difficulties if they answered yes, and if they felt confident to self‐manage their symptoms going forward (yes, no, yes and no).

### Statistical analysis

The data were imported into IBM SPSS Statistics. Each symptom variable was transformed into dichotomous values, allowing data with multiple options to be simplified for analysis (e.g., none or mild were transformed into absence of symptoms and moderate or severe were transformed into presence of symptoms). Stool frequency was transformed into normal (once every 3 days to three times a day) or abnormal (less than once every 3 days or more than three times a day). Stool consistency was transformed into normal (Bristol Stool Form types 3, 4 or 5) or abnormal (Bristol Stool Form types 1, 2, 6 or 7) as previously reported.^20^

*χ* ^2^ tests for independence were used to explore the relationship between two categorical variables between baseline and long‐term follow‐up or across the three‐time points (baseline, short‐term follow‐up and long‐term follow‐up). These tests compared the observed proportions of cases that occurred in each of the categories against the expected values if there was no association between two variables. The Yates Continuity Correction value was used. If the minimum expected cell frequency in any cell was less than 5, Fisher's exact test value was used for greater accuracy. McNemar's test was used to analyse intravariability within patients between different time points. Statistical significance was considered for *p* < 0.05.

## RESULTS

**Figure 1:**
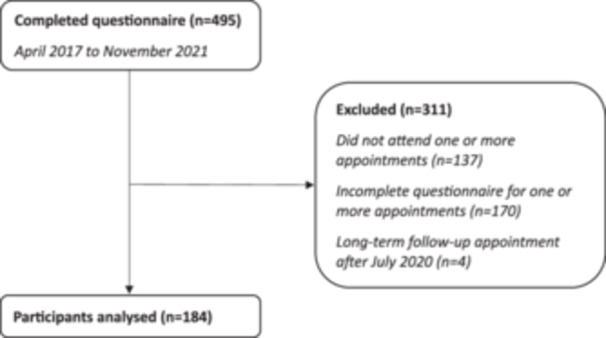
Study flow diagram.

Of 495 patients, 366/495 (74%) were female; the mean (SD) age was 41 (14) years. A complete set of symptom data were available for 184 patients and were eligible to be included in analysis. The remaining 311 patients were excluded because they did not attend one or more appointments (*n* = 137), the questionnaire was incomplete for one or more appointments (*n* = 170), or their long‐term follow‐up appointment was after July 2020 (*n* = 4) (Figure 1).

### Symptoms

For the symptom analysis, 132/184 (72%) were female, the mean (SD) age was 40 (13) years and the mean (SD) body mass index (BMI) was 26.6 (5.4) kg/m^2^ (Figure 1). For GSS, 25/184 (14%) patients reported satisfactory relief from symptoms at baseline, which significantly increased to 127/184 (69%) at short‐term follow‐up and 104/184 (57%) at long‐term follow‐up (*p* < 0.001). Of the patients reporting satisfactory relief at short‐term follow‐up, 85/127 (67%) maintained this in the long term.

**Figure 2:**
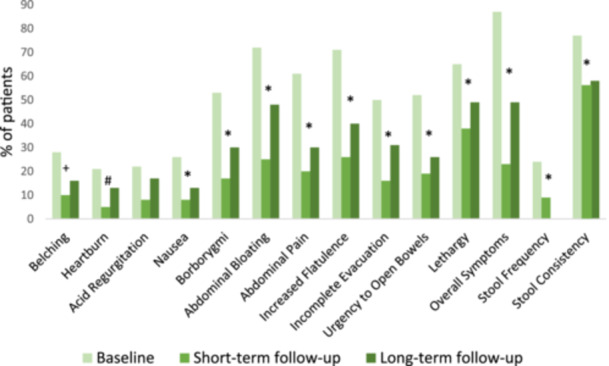
Proportion of patients reporting the presence of symptoms, abnormal stool frequency and abnormal stool consistency according to the Bristol stool form scale at baseline, short‐term follow‐up and long‐term follow‐up (*n* = 184). Statistical analysis was carried out using McNemar's test between baseline and long‐term follow‐up. ^#^ *p* = 0.034; ^+^ *p* = 0.003; *p < 0.001.

Figure 2 shows the proportion of patients who reported individual symptoms, stool frequency and stool consistency at each time point. *χ* ^2^ analysis indicated that the LFD was particularly effective at reducing abdominal bloating [baseline 132/184 (72%) vs. long‐term follow‐up 88/184 (48%; *p* < 0.001)], abdominal pain [baseline 112/184 (61%) vs. long‐term follow‐up 56/184 (30%; *p* < 0.001)] and flatulence [baseline 131/184 (71%) vs. long‐term follow‐up 73/184 (50%; *p* < 0.001)]. The only symptom that did not significantly improve was acid regurgitation, which was reported in a minority of patients at each time point [baseline 40/184 (22%) vs. long‐term follow‐up 31/184 (17%; *p* = 0.150)]. For stool output, there was a significant reduction in the proportion of patients reporting abnormal stool frequency [baseline 43/177 (24%) vs. short‐term follow‐up 15/177 (9%; *p* < 0.001)] (long‐term data were not collected), and there was a significant reduction in the proportion of patients reporting abnormal stool consistency [baseline 134/174 (77%) vs. long‐term follow‐up 101/174 (58%; *p* < 0.001)].

### Adherence and satisfactory symptom relief

The criteria for adherence/non‐adherence are described above and were reported in 176/184 (96%) patients at short‐term follow‐up and in 180/184 (98%) patients at long‐term follow‐up.

At short‐term follow‐up, 166/176 (94%) reported adherence to the restriction phase of the LFD. Adherence did not impact the proportion of patients who reported satisfactory relief of symptoms (*p* = 0.191).

At long‐term follow‐up, 138/180 (77%) reported adherence to FODMAP reintroduction and FODMAP personalisation. Of the patients who had not adhered at long‐term follow‐up, 13/180 (7%) had returned to their habitual diet, 11/180 (6%) were still following FODMAP restriction and 18/180 (10%) were still following FODMAP reintroduction. At long‐term follow‐up, adherence did impact the proportion of patients who reported satisfactory relief of symptoms (*p* = 0.032) (Table 1).

**Table 1:** Proportion of patients reporting adherence with the low‐FODMAP diet and global symptom severity at short‐term and long‐term follow‐up.

Adherence	Global Symptom Severity		*P*
	Unsatisfactory relief	Satisfactory relief	
Short term			
Adherence	42% (*n* = 74/176)	52% (*n* = 92/176)	*p* = 0.191^a^
Nonadherence	1% (*n* = 2/176)	5% (*n* = 8/176)	
Long term			
Adherence	29% (*n* = 53/180)	47% (*n* = 85/180)	*p* = 0.032^b^
Nonadherence	13% (*n* = 24/180)	10% (*n* = 18/180)	

*Note*: Short‐term adherence was defined as FODMAP restriction 75%–100% of the time and non‐adherence was defined as FODMAP restriction less than 75% of the time. Long‐term adherence was defined as following a modified low‐FODMAP diet and non‐adherence was where patients reported that they continued on a strict low‐FODMAP diet, or FODMAP reintroduction or had returned to normal eating.

Abbreviation: FODMAP, fermentable oligosaccharide, disaccharide, monosaccharide and polyol.

^a^ Fisher's test between baseline and short‐term follow‐up.

^b^ *χ* ^2^ test between baseline and long‐term follow‐up.

### Patient experiences at long‐term follow‐up

**Figure 3:**
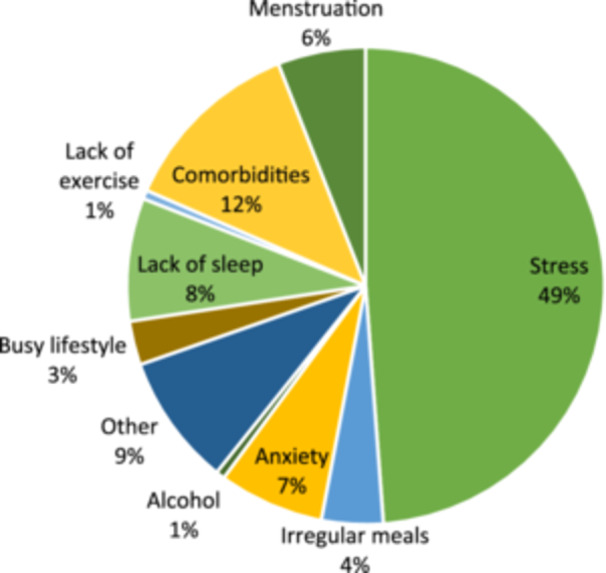
Percentage distribution of nondietary factors reported to contribute to irritable bowel syndrome symptoms.

**Figure 4:**
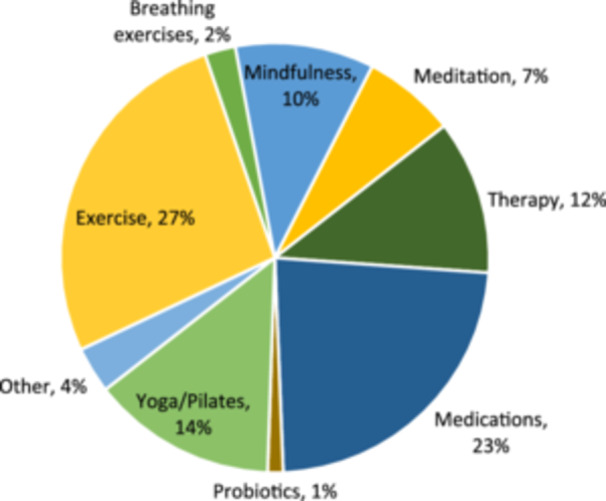
Percentage distribution of nondietary interventions reported to be used to manage irritable bowel syndrome symptoms.

Nondietary factors had contributed to 114/153 (75%) of patients' symptoms, including stress, comorbidities (e.g., diverticulitis), anxiety and lack of sleep (Figure 3). Use of nondietary interventions to help manage symptoms was reported in 57/111 (51%) patients, including medications (e.g., loperamide, omeprazole), yoga/Pilates, exercise (e.g., walking, swimming) and therapy (Figure 4).

Difficulties following the LFD challenges were reported in 73/136 (54%) patients, with 20 patients citing at least one reason: it was hard to follow the challenges (7), hard to fit the challenges into a busy lifestyle (11) and hard to find the length of time it took to complete FODMAP reintroduction (4). The LFD helped with social situations in 47/143 (33%) patients, although 70/143 (49%) patients found that the diet hindered social situations, commonly citing difficulties eating out or being an inconvenience for friends and family. The reintroduction instructions were perceived to be easy to follow in 106/139 (76%) patients; however, 30/139 (22%) patients found them okay and 3/139 (2%) patients found them difficult. Finally, 142/170 (84%) patients felt confident to self‐manage symptoms following the long‐term follow‐up appointment.

### FODMAP tolerance during reintroduction

**Figure 5:**
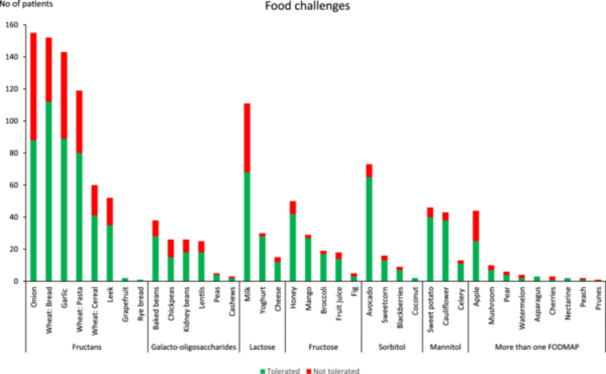
Self‐reported food challenge results at long‐term follow‐up. Patients reported the results of their 3‐day fermentable oligosaccharide, disaccharide, monosaccharide and polyols (FODMAP) food challenges as either ‘tolerated’, meaning that it was not identified as a dietary trigger during the food challenge, or ‘not tolerated’, meaning that it was identified as a dietary trigger during the food challenge. The foods challenged were specific high‐FODMAP foods for patients to challenge as part of the FODMAP reintroduction advice at their short‐term follow‐up appointment.

A subset of patients, 169/184 (92%), included information about which high‐FODMAP foods they had challenged during FODAMP reintroduction and whether these foods were tolerated or not (Figure 5). The data show that 1361 challenges took place, with 965 (71%) challenges being tolerated. The largest proportion of food challenges was for fructan‐containing foods, with 684 challenges and the top 3 food challenges (tolerated/total; % tolerated) were onion (88/155; 57%), wheat bread (112/152; 74%) and garlic (89/143; 62%). Lactose was the second most frequently reported FODMAP group, with 156 food challenges and these comprised milk (68/111; 61%), yoghurt (28/30; 93%) and cheese (12/15; 80%).

## DISCUSSION

This service evaluation describes the long‐term outcomes of patients with IBS who were provided with dietary advice on the LFD in secondary care. Satisfactory relief of symptoms was reported by 57% of patients at long‐term follow‐up, a result consistent with other recent studies investigating the efficacy of the LFD that recorded 55%–89% of patients reporting long‐term satisfactory symptom relief according to local protocol,^12^, ^15^, ^16^, ^21^, ^22^, ^23^, ^24^, ^25^ despite differences in study designs, care settings and cohort sizes. Collectively, these results indicate that the LFD is an effective dietary treatment strategy in IBS in clinical practice. Furthermore, 67% of the patients reporting satisfactory relief at short‐term follow‐up maintained satisfactory relief in the long term, concurrent with 70% reported by O'Keeffe et al., indicating that short‐term symptom improvement is likely to last long term.^12^

Most core individual IBS symptoms improved significantly in the long term when compared to baseline, the only exception being acid regurgitation. This is consistent with other studies that also observed significant positive improvements in most individual symptoms.^12^, ^15^, ^16^ For example, Seamark et al. observed significant improvements in all symptoms, except for acid regurgitation and heartburn; Rej et al. observed significant improvements in all symptoms, except for incomplete evacuation and lethargy; and O'Keeffe et al. observed significant improvements only in abdominal pain, abdominal bloating, flatulence, incomplete evacuation and lethargy. Variations in results may be due to differences in study designs, type of care setting, sample sizes, inclusion criteria and definitions used in recording the data. The greatest improvements in the present study were for abdominal bloating, abdominal pain and flatulence, the presence of which were reported by over 60% of patients at baseline but then decreased by at least a third by long‐term follow‐up. These were also the most reported symptoms at baseline in the three other long‐term studies cited, each reporting significant reduction by long‐term follow‐up. Collectively, these results indicate that the LFD provides a therapeutic long‐term benefit for most individual IBS symptoms and has a significant long‐term benefit in three of the most common symptoms: abdominal bloating, abdominal pain and flatulence.

Studies collecting qualitative data have indicated that patients consider issues with bowel habits to cause the most disruption to their daily lives,^16^ and studies investigating the LFD have reported significant improvements in stool frequency and consistency by long‐term follow‐up.^15^, ^22^, ^26^, ^27^ Likewise, in this study, there was a significant reduction in the proportion of patients reporting abnormal stool consistency by long‐term follow‐up, thus supporting the existing evidence that the LFD improves stool consistency at long‐term follow‐up.

No significant association was found between adherence to the LFD at short‐term follow‐up and satisfactory relief of symptoms. However, long‐term adherence was associated with satisfactory relief of symptoms, which is similar to other findings. O'Keeffe et al. used the same criteria to record long‐term adherence and found similarly significant results, with 72% adherent and reporting satisfactory relief.^12^ Another study also found that those with strict adherence at long‐term follow‐up reported better symptom response.^15^ However, validated scales to measure adherence to the LFD are not yet available,^8^ and studies currently use varying methods of measurement. Comparing the current results with those from other studies, long‐term adherence is significantly associated with global symptom improvement. The degree to which LFD adherence affects symptoms is not yet clear and so future studies may benefit from validated scales to ascertain how strictly adherence must be advocated to influence symptoms.

Understandably, some patients may be hesitant to complete FODMAP reintroduction when FODMAP restriction has improved IBS symptoms. However, there are nutritional implications associated with restrictive diets in the long term.^11^, ^18^ Dietary diversity can be increased by reintroducing high‐FODMAP foods to tolerance, and a modified LFD has been shown to be nutritionally adequate and acceptable to follow and not negatively impact the microbiome in the long term.^12^, ^28^ In our study, 77% of patients reported adherence to the LFD as instructed at long‐term follow‐up and improvement with GSS. This is consistent with other studies of similar size,^15^ indicating that the reintroduction of high‐FODMAP foods to tolerance does not increase symptoms. Moreover, FODMAP reintroduction should be actively encouraged to increase dietary variety, thereby reducing the risk of nutritional deficiencies and disruptions to the microbiome.^28^, ^29^

A few studies have reported on the tolerance of high‐FODMAP foods during FODMAP reintroduction. It is interesting that the current study reports that over two‐thirds of food challenges are tolerated. This provides some data to reassure people undertaking FODMAP reintroduction who are concerned about exacerbating symptoms during food challenge that foods not tolerated are in the minority. Research in 2053 users of an LFD app supports these findings and during FODMAP reintroduction, symptoms were only triggered in 35%–41% of users for the five top foods challenged; these were wheat bread, onion, garlic, milk and wheat pasta.^30^ The current study also identified the same foods as the most commonly reported food challenges during FODMAP reintroduction. These data provide an impression of how different foods and FODMAP groups are tolerated during the reintroduction phase, further study of which could influence further development of the low‐FODMAP diet and its education.

Overall, patients found that the reintroduction instructions were easy to follow and felt confident in self‐managing symptoms going forward, indicating that the resources and counselling provided in clinical practice were effective. However, over half of patients reported difficulties with following the diet, such as fitting it into a busy lifestyle, and just under half found that the diet hindered social situations, consistent with other studies that have reported that following the LFD can cause disruption for patients when eating out with family and friends.^12^

The strengths of this study include its large cohort size, the extent of data captured and the reduced risk of nonresponse bias by completing questionnaires during appointments. Furthermore, all FODMAP advice was provided by dietitians experienced in educating on the LFD as part of routine clinical practice. This ensured that the information provided was safe, consistent, evidence‐based and that patients were monitored appropriately. However, there are several limitations. First, this was an observational service evaluation. This can introduce bias due to the lack of blinding and randomisation, social desirability or recall, potentially high rates of patients not returning for review if their symptoms did not improve and incomplete questionnaires, the latter of which resulted in 307 individuals being excluded from the study (some of whom missed only one or two questions).

Questionnaires were completed on paper and data were gathered manually by several different researchers, with an error rate of 3%, indicating that the accuracy of the data set may have been compromised by interoperator variation and erroneous data entry. This could be improved with electronic form‐based data capture, which would eliminate errors introduced through intermediate interpretation or data entry, while also improving data hygiene through the use of input validation to ensure that the data are complete before submitting. Another limitation is that some of the symptom improvements in this study may not be solely attributable to the LFD. Seventy‐five percent of patients believed that nondietary factors contributed to their symptoms, such as stress or comorbidities, limiting the impact of dietary intervention. Fifty‐three percent of patients used nondietary interventions to help manage symptoms, such as medications, which could have a confounding impact on results. Accordingly, randomised‐controlled trials that take these factors into account would be needed to determine the extent of the improvements that can be attributable to the LFD. Finally, this sample may not be representative of the entire IBS population, as data were only gathered from one secondary care service in an urban setting. Referrals to a teaching hospital may include more complex IBS cases with more severe symptoms, and the results may differ significantly if the study were to be repeated in a primary care population. The ratio between male and female participants was similar to IBS prevalence data,^31^ however, the results may not be generalisable to patients in primary care or to populations in rural settings.

This study reinforces existing knowledge about the long‐term effect of the LFD on IBS symptoms. Additionally, it provides a novel insight into the benefits of long‐term adherence with the diet and reintroduction of high‐FODMAP foods as instructed, which can be applied to low‐FODMAP counselling in dietetic services. However, a large proportion of the cohort was excluded for not attending all three of their appointments. Further research could investigate why a large proportion of patients do not complete the LFD education.

## CONCLUSION

This service evaluation showed that the LFD was clinically effective at providing satisfactory long‐term relief from IBS symptoms in nearly two‐thirds of patients, which is in line with the current literature. For individual symptoms, the LFD is a, particularly, effective therapy for reducing abdominal bloating, abdominal pain and flatulence. Better symptom improvement is seen when people adhere with the diet in the long term and when reintroduction of high‐FODMAP foods to tolerance is completed as instructed. For patients who challenged with high‐FODMAP foods, more than two‐thirds of food challenges were well tolerated. High‐ quality randomised‐controlled trials in primary and secondary care are required to explore the cause and effect relationship between the LFD and IBS symptom management.

## AUTHOR CONTRIBUTIONS

Miranda Lomer designed the research. Lara Dami, Paru Shah, Alice Twomey, Danielle Jones and Rosie Foulkes collected the data. Rosie Foulkes processed the data, performed the statistical analysis and designed the tables and figures. Rosie Foulkes wrote the manuscript. All authors reviewed and commented on the final manuscript.

## CONFLICT OF INTEREST STATEMENT

Miranda Lomer leads postregistration courses for dietitians on the dietary management of irritable bowel syndrome. The remaining authors declare no conflict of interest.

## PEER REVIEW

The peer‐review history for this article is available at https://www.webofscience.com/api/gateway/wos/peer-review/10.1111/jhn.13393.

## Data Availability

The data that support the findings of this study are available on request from the corresponding author. The data are not publicly available due to privacy or ethical restrictions.
